# Feasibility of Training Family Caregivers of People Living with Dementia Online to Deliver Life Review Depression Intervention at Home

**DOI:** 10.1002/alz.088251

**Published:** 2025-01-09

**Authors:** Christina E. Miyawaki, Erin D. Bouldin, Angela McClellan

**Affiliations:** ^1^ University of Houston Graduate College of Social Work, Houston, TX USA; ^2^ University of Utah School of Medicine, Salt Lake City, UT USA

## Abstract

**Background:**

Due to the high prevalence of depressive symptoms and dementia in older Americans (≥65 years), we trained unpaid family caregivers in an evidence‐based life review depression intervention virtually via Zoom and produced positive outcomes. The objective of this study was to examine the feasibility of training caregivers via online video and delivering the intervention at home.

**Method:**

We recruited caregiver‐care recipient dyads nationwide during the COVID‐19 pandemic (N = 20 dyads). A mixed‐methods design was used to examine the results quantitatively and supported the findings with qualitative interviews with caregivers. We used fidelity scores as a measure of caregivers’ feasibility to follow the intervention protocol and conduct the life reviews. We collected the pre‐ and post‐intervention measures on care recipients’ depression as the primary outcome, and life satisfaction, caregivers’ caregiving burden, rewards of caregiving, and dyads’ relationship quality as secondary outcomes, and analyzed them using paired t‐tests.

**Result:**

Caregivers were on average 64 years old, White (65%), and female (90%). Many were married (65%), college‐educated (70%), working or retired (40% each), and in good‐excellent health (100%). Their care recipients were 80 years old (mean), and White (70%). Gender and their health conditions were equally divided into male and female (50% each), and in poor‐fair and good‐excellent health (50% each). The online‐trained caregivers’ average fidelity check‐in score was 15.86±0.268 (out of 16), which was a significant improvement from that of Zoom‐trained caregivers (14.83±0.776, *p*<0.001) indicating excellent adherence to the intervention protocol. The C‐PLR intervention significantly positively impacted care recipients’ depressive symptoms (*p* = 0.034), as well as caregivers’ rewards of caregiving (*p* = 0.019), especially care recipients who lived alone (*p* = 0.024). Caregivers’ post‐intervention qualitative interviews aligned with the quantitative results supporting that online‐trained caregivers could deliver the intervention by the protocol without adding caregiver burden.

**Conclusion:**

The outcomes of the study showed that family caregivers could learn life review skills through online videos and perform life reviews closely adhering to the study protocol. This study demonstrated that the *Caregiver‐Provided Life Review*, an innovative, cost‐effective intervention with a flexible delivery schedule could make a positive impact on both caregivers’ and care recipients’ mental health.

